# Consumption of ultra-processed foods and risk of all-cause and cause-specific mortality: the Singapore Chinese health study

**DOI:** 10.1186/s12937-025-01219-0

**Published:** 2025-09-29

**Authors:** Yue Li, Xianli Li, An Pan, Woon-Puay Koh

**Affiliations:** 1 Department of Epidemiology and Biostatistics, Ministry of Education Key Laboratory of Environment and Health, School of Public Health, Tongji Medical College, Huazhong University of Science and Technology, Wuhan, Hubei Province China; 2 Healthy Longevity Translational Research Programme, Yong Loo Lin School of Medicine, National University of Singapore, Singapore, Singapore; 3 Institute for Human Development and Potential (IHDP), Agency for Science, Technology and Research (A*STAR), Singapore, Singapore; 4 Centre for Obesity and Diabetes Research, School of Public Health, Tongji Medical College, Huazhong University of Science and Technology, Wuhan, Hubei Province,, China

**Keywords:** Asian population, Epidemiology, Mortality, Nutrition, Ultra-processed food

## Abstract

**Background:**

Although higher intake of ultra-processed foods (UPFs) has been associated with a higher risk of mortality in Western populations such as populations from France and the United States (US), evidence in Asian populations remains limited. The aim of this study was to evaluate the association between UPF consumption and the risk of mortality in an Asian population.

**Methods:**

We included 62,197 middle-aged and older Chinese adults who were recruited for the Singapore Chinese Health Study from 1993 to 1998. UPFs were defined from items in the FFQ using the Nova classification, and their consumption was categorized into quintiles according to intake level. Mortality from all-cause, cardiovascular diseases (CVDs), cancer, and respiratory diseases were ascertained via Linkage with a nationwide registry through 2022. Associations between UPF intake and mortality were assessed using Cox proportional hazards regression models.

**Results:**

After 24.9 years (median) of follow-up, 29,472 deaths occurred. In the multivariable-adjusted model (variables related to demographics, anthropometric data, lifestyle factors, medical history, and total energy intake), compared with the lowest quintile of UPF consumption, the highest quintile was associated with higher risks of mortality from all-cause [hazard ratio (HR): 1.06; 95% confidence interval (CI): 1.02–1.10], CVDs (HR: 1.08; 95% CI: 1.01–1.15), and respiratory diseases (HR: 1.10; 95% CI: 1.02–1.19), but not of mortality from cancer (HR: 1.00; 95% CI: 0.94–1.07). The associations remained essentially unchanged after further adjusting for diet quality measured using the Alternative Healthy Eating Index-2010 and antioxidant capacity using the Vitamin C Equivalent Antioxidant Capacity. Among the subgroups of UPFs, positive associations with all-cause mortality were observed for consumption of sweetened beverages (e.g. soft drinks) and sugary products (e.g. crackers and western cakes). This association was stronger in participants who were non-smokers at recruitment [respective HR: 1.08, 95% CI: 1.03–1.13 in non-smokers versus HR: 1.01; 95% CI: 0.94–1.08 in smokers (*P* for interaction = 0.03)].

**Conclusion:**

Higher intake of UPFs was associated with higher risks of mortality from all-cause, CVDs, and respiratory diseases in an Asian population. These results need to be confirmed in other Asian populations.

**Supplementary Information:**

The online version contains supplementary material available at 10.1186/s12937-025-01219-0.

## Introduction

Ultra-processed foods (UPFs) are industrial formulations that typically include ingredients such as preservatives, sweeteners, colorings, flavorings, and emulsifiers, and which are manufactured from a series of industrial processes (e.g. hydrogenation and extrusion) [1]. These added ingredients in UPFs are generally cosmetic additives (e.g. emulsifiers) that enhance their texture and appearance, and are not commonly used in culinary preparations [1]. Being affordable and highly palatable, these foods are becoming dominant in the global food system [2]. In some high-income countries like the United States (US) and United Kingdom (UK), UPF consumption accounts for more than 50% of daily energy intake [2, 3]. In Asian countries, increasing industrialization, globalization, and technological change of food systems have also contributed to the increasing consumption of UPFs in the Asian region [4]. For example, in Korea, the contribution of UFPs to daily energy intake has increased from 17.41 to 25.33% between 1998 and 2022 [5].

Unfortunately, UPFs are often high in added sugars, sodium, and saturated fats, and low in fiber [6]. In addition, UPFs may contain potentially toxic compounds, such as additives (e.g. low-calorie sweeteners), contaminants formed during the processing (e.g. acrylamide), and substances migrating from contact packaging (e.g. bisphenols) [7–9]. A meta-analysis showed that higher UPF consumption was associated with a higher risk of mortality [10]. However, studies included in this meta-analysis were all conducted in Western populations (e.g. populations from Spain, the US, and France). It is known that traditional dietary patterns in Asian populations have distinct differences from Western populations in Europe and North America [11, 12]. Yet, to date, only one Asian study has investigated the association between UPF intake and mortality, and although this study, conducted in Korea, found that ultra-processed red meat and fish in both sexes, and milk and soymilk drinks in male were positively associated with all-cause mortality, it did not report an association between total UPF intake and all-cause, cancer or cardiovascular disease (CVD) mortality [13]. Hence, more studies are needed to investigate the impact of UPFs in Asian populations.

To fill these knowledge gaps, we aimed to investigate the association between UPF intake and the risk of all-cause and cause-specific mortality among middle-aged and older Chinese living in Singapore.

## Methods

### Study population

The Singapore Chinese Health Study (SCHS) is a population-based cohort study of Chinese adults in Singapore. From 1993 to 1998, 63,257 adults aged 45–74 years were recruited; they were all permanent residents or citizens of Singapore from two major dialect groups, i.e. the Hokkien or Cantonese, who originated from the Fujian or Guangdong province in the southern part of China, respectively [14]. For our study, we excluded participants with implausible energy intake (male with < 700 kcal/d or > 3700 kcal/d or female with < 600 kcal/d or > 3000 kcal/d) [15, 16], leaving 62,197 participants for analysis (Supplementary Fig. 1). This study was approved by the Institutional Review Board of the National University of Singapore. All participants provided written consent.

### Dietary assessment

Habitual dietary intakes over the past 12 months were collected through a 165-item, semi-quantitative food frequency questionnaire (FFQ) by trained interviewers at baseline. The FFQ was developed specifically for this cohort and subsequently validated against two 24-h recalls within a subcohort of participants from SCHS [14]. Participants were asked to choose from eight predefined frequencies (ranging from never or hardly ever to ≥ 2 times/day) and serving sizes (generally three options including small, medium, and large) for each food item or dish. Daily energy and nutrient intakes were calculated using the Singapore Food Composition Database specifically developed for this cohort [14].

Nova food classification was applied to selected items from the FFQ, grouping food and beverage items into the following four categories according to the purpose and extent of food processing: (1) unprocessed or minimally processed foods (e.g. fruit and vegetables); (2) processed culinary ingredients (e.g. sugar and butter); (3) processed foods (e.g. canned fish); (4) UPFs (e.g. soft drinks and processed meat) [1]. UPFs were identified based on two important characteristics: industrial substances not commonly used in kitchens and additives used to make them palatable or more appealing [1]. Hard liquor is classified as UPFs, and alcohol is a well-investigated risk factor for mortality; therefore, we did not consider hard liquor in UPFs in our primary analysis. In addition, since whole-wheat bread, which is classified as UPFs, has been associated with lower ischemic heart disease mortality in our study population [17], we also did not include whole-wheat bread in UPFs in the primary analysis. Details of UPFs along with some examples are presented in Supplementary Table 1. For each participant, UPF consumption was evaluated by dividing the weight of UPF consumption (g/day, the sum of each food item in the fourth category of the Nova classification) by the weight of total food consumption (g/day). In our analysis, we considered weight ratio rather than energy ratio because the former better accounts for the non-nutritional factors related to food processing (e.g. additives and neo-formed contaminants) [18–20].

### Outcome ascertainment

Deaths from all-cause, CVD, cancer, and respiratory diseases were identified via Linkage with the nationwide registry of births and deaths in Singapore through December 31, 2022. Due to the transition from ICD-9 to ICD-10 in the Registry of Births and Deaths in Singapore, the International Classification of Diseases ninth (ICD-9, up to 31 December 2011) or tenth (ICD-10, from 2012 to 2022) revision codes were used to classify causes of death from CVD (390–459 or I00–I99), cancer (140–208, C00–C97), and respiratory diseases (460–519 or J00–J99).

### Covariate assessment

Information on demographics (age, sex, education, and dialect group), anthropometric data (height and weight), lifestyle factors (smoking status, alcohol frequency, sleep duration, and physical activity), and medical history (hypertension, coronary artery disease, stroke, diabetes, and cancer) were collected at baseline using a structured questionnaire. History of cancer was further ascertained via the linkage with the database of the nationwide Singapore Cancer Registry. Body mass index (BMI) was calculated as weight (kg) divided by the square of the height in meters.

The Alternative Healthy Eating Index (AHEI)−2010 score in our study was computed from 10 components of whole grains, vegetables, fruits, sugar-sweetened beverages and fruit juice, nuts and legumes, red meat, long-chain n–3 fatty acids, polyunsaturated fatty acids (PUFAs), sodium, and alcohol (trans fat not including due to very low consumption level in Singapore and lack of information on intake level in this cohort) [21]. The Vitamin C Equivalent Antioxidant Capacity (VCEAC) was based on the database of Vitamin C Equivalents (VCE) constructed by Floegel et al. and considered the daily intake levels of 12 antioxidants (i.e., 5 categories of carotenoids, 5 categories of flavonoids, vitamin C, and vitamin E) [22]. Both the AHEI-2010 and VCEAC have been associated with risk of mortality and other chronic diseases in this SCHS cohort [23–26].

### Statistical analysis

Continuous variables were shown as mean ± SD or median [interquartile range (IQR)], and categorical variables were expressed as numbers (%). Follow-up time was calculated from the date of recruitment until the recorded date of death, loss to follow-up, or December 31, 2022, whichever came first. Cox proportional hazard regression models were used to estimate the hazard ratios (HRs) and corresponding 95% confidence intervals (CIs) for the associations between the weight ratio of UPFs (as sex-specific quintiles or continuous variable rescaled as per 10%) and the risk of mortality. The proportional hazards assumption of the Cox models was confirmed by the Schoenfeld residuals method and inspected time-based plots. Linear trends were tested by assigning the median proportion of UPFs within each quintile to participants of that specific quintile and treating this as a continuous variable. Restricted cubic spline (RCS) analysis with three knots (10th, 50th, and 90th percentiles) was used to examine the dose–response association between UPF intake as a continuous variable (per 1% in the weight ratio) and the risk of mortality, and the linearity was tested by Wald 𝜒^2^ tests.

In Model 1, we adjusted for age (continuous, years), sex (male or female), total energy intake (continuous, kcal/d), dialect group (Cantonese or Hokkien, to account for possible genetic, dietary, and lifestyle differences between Hokkiens and Cantonese), educational level (no formal education, primary school, or secondary school or higher), body mass index (< 18.5, 18.5–22.9, 23.0-27.4, 27.5 + kg/m^2^), smoking status (never, former, or current), alcohol frequency (none, monthly, weekly, or daily), physical activity (< 0.5, 0.5 to 3.9, or ≥ 4.0 h/wk), and sleep duration (< 6, 6 to 8, or > 8 h/d). In Model 2, we additionally adjusted for the history of hypertension, diabetes, CVDs (coronary artery disease or stroke), and cancer. To test for the potential confounding influence of quality and antioxidant capacity of diet measured by AHEI-2010 or VCEAC, we first investigated the correlations between UPF intake and these diet scores using the Spearman correlation coefficients. Subsequently, we investigated the association between UPF intake and the risk of mortality with the adjustment for these two diet scores respectively. Finally, we also investigated the associations between the top four most highly consumed subgroups of UPFs and the risk of mortality. There were 134 participants (0.2%) who had missing values for VCEAC, and we excluded them from the analyses that included VCEAC as covariates.

Stratified analyses were conducted by selected factors at recruitment: age (≤ 60, > 60 y), sex (male, female), BMI (< 23, ≥ 23 kg/m^2^), physical activity (< 0.5, 0.5–3.9, ≥ 4 h/week), smoking status at recruitment (non-smoker, smoker), history of hypertension (no, yes), and history of diabetes (yes, no). Potential interaction was tested using the Wald test by adding relevant product terms in Model 2.

Several sensitivity analyses were conducted to test the robustness of our results. First, we excluded participants who died within 2 or 3 years or participants with history of CVD or cancer at baseline. Second, according to the standard Nova classification, we added back to UPFs whole-wheat bread and hard liquor individually or in combination and repeated our analysis. Third, we used quintiles based on the whole cohort to assess the association between UPF intake and mortality. Finally, we investigated the association between the absolute intake of UPFs (g/day) and the risk of mortality.

All analyses were performed using SAS version 9.4 (SAS Institute) and R software (version 4.2.3). Two-sided *P* < 0.05 was considered to be statistically significant.

## Results

### Participant characteristics

Of 62,197 participants included in our analysis, the mean age was 57.0 ± 8.0 years, and 34,719 (55.8%) were female. Participants with higher UPF intake were more likely to be non-smoking, non-drinking, and to have higher energy intake and lower scores of AHEI-2010 than those with lower UPF intake (Table 1). The median weight proportion of UPFs in the diet was 6.1%. Of UPF subgroups, sweetened beverages (53.7%), cereals and starchy foods (30.0%), sugary products (5.5%), and ultra-processed meat, fish, and eggs (4.5%) contributed most to the overall UPF intake (Supplementary Fig. 2).

**Table 1:** Baseline characteristics according to quintiles of ultra-processed food consumption among individuals in the Singapore Chinese health study

	Sex-specific quintiles of ultra-processed food consumption^a^				
	Q1	Q2	Q3	Q4	Q5
Weight ratio, Median [IQR]	1.1 (0.5–1.6)	3.3 (2.7–3.9)	6.1 (5.3-7.0)	11.0 (9.5–12.9)	20.3 (17.3–24.8)
Number of subjects	12,438 (20.0)	12,440 (20.0)	12,440 (20.0)	12,440 (20.0)	12,439 (20.0)
Age, years	57.2 ± 7.8	56.7 ± 7.9	56.5 ± 8.0	56.5 ± 8.0	57.9 ± 8.3
Male	5495 (44.2)	5496 (44.2)	5496 (44.2)	5496 (44.2)	5495 (44.2)
Dialect group					
Cantonese	5803 (46.7)	5926 (47.6)	5865 (47.1)	5704 (45.9)	5573 (44.8)
Hokkien	6635 (53.3)	6514 (52.4)	6575 (52.9)	6736 (54.1)	6866 (55.2)
Educational level					
No formal education	3775 (30.4)	3402 (27.3)	3197 (25.7)	3056 (24.6)	3501 (28.1)
Primary school	5757 (46.3)	5635 (45.3)	5411 (43.5)	5492 (44.1)	5356 (43.1)
Secondary school or higher	2906 (23.4)	3403 (27.4)	3832 (30.8)	3892 (31.3)	3582 (28.8)
Body mass index group					
Underweight [< 18.5 kg/m^2^]	698 (5.6)	761 (6.1)	777 (6.2)	848 (6.8)	914 (7.3)
Normal weight [18.5 to 22.9 kg/m^2^]	4901 (39.4)	5121 (41.2)	5277 (42.4)	5293 (42.5)	5310 (42.7)
Overweight [23.0 to 27.4 kg/m^2^]	5607 (45.1)	5455 (43.9)	5330 (42.8)	5275 (42.4)	5277 (42.4)
Obese [≥ 27.5 kg/m^2^]	1232 (9.9)	1103 (8.9)	1056 (8.5)	1024 (8.2)	938 (7.5)
Smoking status					
Never	8043 (64.7)	8436 (67.8)	8848 (71.1)	8894 (71.5)	9015 (72.5)
Former	1305 (10.5)	1357 (10.9)	1380 (11.1)	1349 (10.8)	1488 (12.0)
Current	3090 (24.8)	2647 (21.3)	2212 (17.8)	2197 (17.7)	1936 (15.6)
Alcohol frequency					
None	9815 (78.9)	9981 (80.2)	10,011 (80.5)	10,089 (81.1)	10,659 (85.7)
Monthly	701 (5.6)	961 (7.7)	1063 (8.6)	969 (7.8)	808 (6.5)
Weekly	1123 (9.0)	1048 (8.4)	1038 (8.3)	1045 (8.4)	759 (6.1)
Daily	799 (6.4)	450 (3.6)	328 (2.6)	337 (2.7)	213 (1.7)
Physical activity					
< 0.5 h/wk	8688 (69.9)	8469 (68.1)	8255 (66.4)	8024 (64.5)	8285 (66.6)
0.5–3.9 h/wk	2213 (17.8)	2457 (19.8)	2576 (20.7)	2560 (20.6)	2466 (19.8)
≥ 4 h/wk	1537 (12.4)	1514 (12.2)	1609 (12.9)	1856 (14.9)	1688 (13.6)
Sleep duration					
< 6 h/d	1303 (10.5)	1137 (9.1)	1061 (8.5)	1183 (9.5)	1301 (10.5)
6–8 h/d	10,257 (82.5)	10,416 (83.7)	10,585 (85.1)	10,412 (83.7)	10,239 (82.3)
> 8 h/d	878 (7.1)	887 (7.1)	794 (6.4)	845 (6.8)	899 (7.2)
History of hypertension	2943 (23.7)	2964 (23.8)	2875 (23.1)	2949 (23.7)	3065 (24.6)
History of diabetes	1230 (9.9)	1119 (9.0)	998 (8.0)	1050 (8.4)	1176 (9.5)
History of cardiovascular disease	584 (4.7)	621 (5.0)	574 (4.6)	643 (5.2)	918 (7.4)
History of cancer	312 (2.5)	368 (3.0)	362 (2.9)	379 (3.0)	478 (3.8)
Dietary factors					
Total energy intake, kcal/d	1397.6 ± 485.5	1518.4 ± 506.3	1573.0 ± 518.3	1673.8 ± 544.1	1565.0 ± 497.3
AHEI-2010	50.3 ± 7.1	50.4 ± 6.7	50.2 ± 6.9	50.0 ± 7.6	49.2 ± 8.1
VCEAC, Median [IQR]	217.8 (101.7-546.4)	228.9 (115.7-515.7)	231.9 (119.8-479.4)	250.7 (129.5-501.7)	181.5 (95.9-355.1)

Values are n (%) for categorical variables and means ± standard deviations for continuous variables unless otherwise specified

*IQR* Interquartile range, *AHEI* Alternative Healthy Eating Index, *VCEAC* Vitamin C Equivalent Antioxidant Capacity

^a^ Quartiles of the weight ratio of ultra-processed food intake in the total food consumed (%). Sex-specific cut-offs for quartiles of ultra-processed weight ratio were 1.8, 3.9, 7.1, and 13.5 in male and 2.6, 5.1, 8.9, and 16.2 in female

### Associations between UPF intake and mortality

After a median follow-up of 24.9 years, 29,472 deaths occurred in this cohort, including 9,322 CVD deaths, 9,259 cancer deaths, and 6,452 respiratory disease deaths. The RCS curve showed positive Linear dose-response relationships between a 1% increase in the weight proportion of UPFs and risks of all-cause mortality, CVD mortality, and respiratory disease mortality (*P* for the overall association: <0.001, 0.002, and < 0.001, respectively; *P* for nonlinearity: 0.36, 0.47, and 0.98, respectively; Supplementary Fig. 3). In the fully adjusted model, compared with participants with the lowest quintile of UPF intake, the HRs (95% CIs) for participants with the highest quintile of UPF intake were 1.06 (1.02, 1.10) for all-cause mortality, 1.08 (1.01, 1.15) for CVD mortality, and 1.10 (1.02, 1.19) for respiratory disease mortality, but not significant for cancer mortality (HR: 1.00; 95% CI: 0.94–1.07) (Table 2). For per 10% increment in the weight proportion of UPFs, the HRs (95% CIs) were 1.04 (1.02, 1.05) for all-cause mortality, 1.04 (1.02, 1.07) for CVD mortality and 1.06 (1.03, 1.09) for respiratory disease mortality.

**Table 2:** Hazard ratios (95% confidence intervals) for associations of ultra-processed food intake with mortality in the Singapore Chinese health study

	Sex-specific quintiles of ultra-processed food consumption, %						
	Q1	Q2	Q3	Q4	Q5	*P* trend	Continuous^a^
Median (IQR)	1.1 (0.5–1.6)	3.3 (2.7–3.9)	6.1 (5.3-7.0)	11.0 (9.5–12.9)	20.3 (17.3–24.8)		
All-cause mortality							
Cases/person-years	6174/270,983	5758/275,330	5606/275,561	5641/274,395	6293/262,751		
Model 1^b^	1 (ref)	0.99 (0.95–1.02)	1.01 (0.98–1.05)	1.04 (1.00-1.08)	1.07 (1.03–1.11)	< 0.001	1.04 (1.02–1.05)
Model 2^c^	1 (ref)	0.98 (0.94–1.01)	1.01 (0.97–1.05)	1.03 (0.99–1.07)	1.06 (1.02–1.10)	< 0.001	1.04 (1.02–1.05)
CVD mortality							
Cases/person-years	1939/270,983	1810/275,330	1716/275,561	1792/274,395	2065/262,751		
Model 1^b^	1 (ref)	0.99 (0.92–1.05)	0.98 (0.92–1.05)	1.04 (0.98–1.12)	1.09 (1.02–1.16)	< 0.001	1.05 (1.02–1.07)
Model 2^c^	1 (ref)	0.96 (0.90–1.03)	0.97 (0.91–1.04)	1.03 (0.96–1.10)	1.08 (1.01–1.15)	< 0.001	1.04 (1.02–1.07)
Cancer mortality							
Cases/person-years	2065/270,983	1862/275,330	1836/275,561	1710/274,395	1845/262,751		
Model 1^b^	1 (ref)	0.99 (0.93–1.05)	1.03 (0.97–1.10)	0.97 (0.91–1.04)	1.02 (0.96–1.09)	0.66	1.01 (0.98–1.04)
Model 2^c^	1 (ref)	0.98 (0.92–1.05)	1.02 (0.96–1.09)	0.96 (0.90–1.03)	1.00 (0.94–1.07)	0.93	1.00 (0.98–1.03)
Respiratory disease mortality							
Cases/person-years	1275/270,983	1250/275,330	1234/275,561	1286/274,395	1407/262,751		
Model 1^b^	1 (ref)	1.02 (0.95–1.11)	1.07 (0.99–1.16)	1.13 (1.04–1.22)	1.11 (1.03–1.20)	0.002	1.06 (1.03–1.09)
Model 2^c^	1 (ref)	1.01 (0.94–1.10)	1.06 (0.98–1.15)	1.12 (1.04–1.22)	1.10 (1.02–1.19)	0.003	1.06 (1.03–1.09)

*IQR* Interquartile range, *CVD* Cardiovascular disease

^a^ Hazard ratio for per increase of 10% in the proportion of ultra-processed food intake

^b^ Model 1: adjusted for age (continuous), sex (male or female), total energy intake (continuous), dialect group (Cantonese or Hokkien), educational level (no formal education, primary school, or secondary school or higher), body mass index (< 18.5, 18.5–22.9, 23.0-27.4, 27.5 + kg/m^2^), smoking status (never, former, or current), alcohol frequency (none, monthly, weekly, or daily), physical activity (< 0.5 h/wk, 0.5–3.9 h/wk, or ≥ 4 h/wk), and sleep duration (< 6 h/d, 6–8 h/d, or > 8 h/d)

^c^ Model 2: Model 1 + history of hypertension (yes/no), history of diabetes (yes/no), history of cardiovascular disease (yes/no), and history of cancer (yes/no)

The UPF intake had a low correlation with AHEI-2010 and VCEAC (respective Spearman correlation coefficients: −0.03 and − 0.07; Table 3). For the risk estimates, the association of UPF intake with all-cause and cause-specific mortality remained materially unchanged after the adjustment of AHEI-2010 or VCEAC in the model.

**Table 3:** Associations of ultra-processed food intake with mortality adjusted for dietary factors.^a^

	Correlation coefficient with ultra-processed food intake	HR (95% CI) for Q1	HR (95% CI) for Q5	*P* trend	Continuous^b^
All-cause mortality					
Model 2		1 (ref)	1.06 (1.02–1.10)	< 0.001	1.04 (1.02–1.05)
Model 2 plus AHEI-2010	−0.03	1 (ref)	1.05 (1.01–1.09)	< 0.001	1.03 (1.02–1.04)
Model 2 plus VCEAC^c^	−0.07	1 (ref)	1.05 (1.01–1.09)	< 0.001	1.03 (1.02–1.05)
CVD mortality					
Model 2	-	1 (ref)	1.08 (1.01–1.15)	< 0.001	1.04 (1.02–1.07)
Model 2 plus AHEI-2010	−0.03	1 (ref)	1.07 (1.00-1.14)	0.002	1.04 (1.01–1.06)
Model 2 plus VCEAC^c^	−0.07	1 (ref)	1.07 (1.00-1.14)	0.001	1.04 (1.01–1.07)
Cancer mortality					
Model 2	-	1 (ref)	1.00 (0.94–1.07)	0.93	1.00 (0.98–1.03)
Model 2 plus AHEI-2010	−0.03	1 (ref)	1.00 (0.94–1.06)	0.81	1.00 (0.98–1.03)
Model 2 plus VCEAC^c^	−0.07	1 (ref)	1.00 (0.94–1.07)	0.97	1.00 (0.98–1.03)
Respiratory disease mortality					
Model 2		1 (ref)	1.10 (1.02–1.19)	0.003	1.06 (1.03–1.09)
Model 2 plus AHEI-2010	−0.03	1 (ref)	1.10 (1.01–1.18)	0.006	1.06 (1.03–1.09)
Model 2 plus VCEAC^c^	−0.07	1 (ref)	1.09 (1.00-1.17)	0.01	1.05 (1.02–1.09)

*HR* Hazard ratio, *CI* Confidence interval, *CVD* Cardiovascular disease, *AHEI* Alternative Healthy Eating Index, *VCEAC* Vitamin C Equivalent Antioxidant Capacity

^a^ Model 2 was adjusted for age (continuous), sex (male or female), total energy intake (continuous), dialect group (Cantonese or Hokkien), educational level (no formal education, primary school, or secondary school or higher), body mass index (< 18.5, 18.5–22.9, 23.0-27.4, 27.5 + kg/m^2^), smoking status (never, former, or current), alcohol frequency (none, monthly, weekly, or daily), physical activity (< 0.5 h/wk, 0.5–3.9 h/wk, or ≥ 4 h/wk), sleep duration (< 6 h/d, 6–8 h/d, or > 8 h/d), history of hypertension (yes/no), history of diabetes (yes/no), history of cardiovascular disease (yes/no), and history of cancer (yes/no)

^b^ Hazard ratio for per increase of 10% in the proportion of ultra-processed food intake

^c^ 134 participants were not included in these analyses because of missing values of Vitamin C Equivalent Antioxidant Capacity

**Fig. 1:**
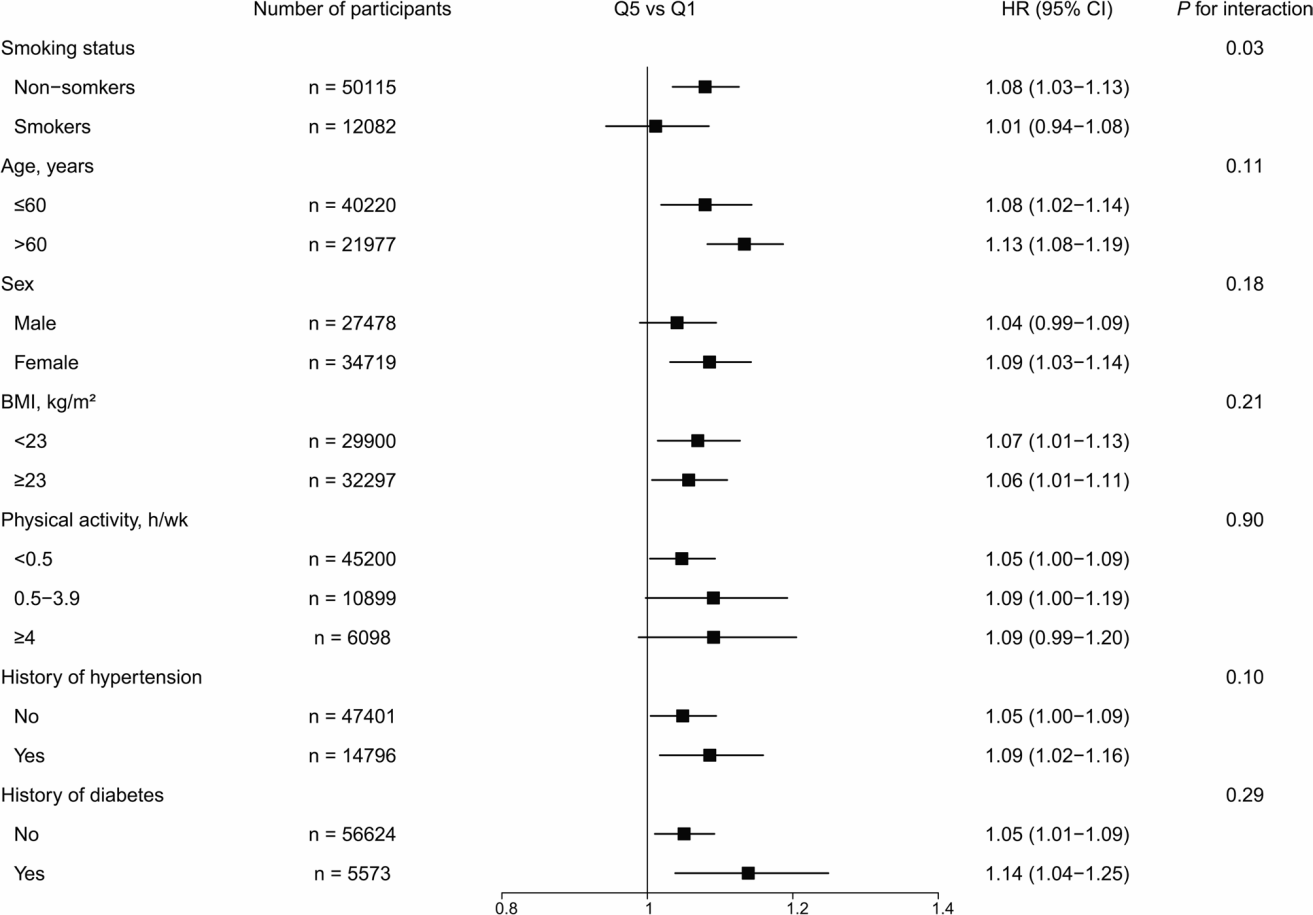
Associations between ultra-processed food intake and all-cause mortality in different strata in the Singapore Chinese Health Study. HR indicates hazard ratio. CI indicates confidence interval. BMI indicates body mass index. Multivariable adjusted models were adjusted for age (continuous), sex (male or female), total energy intake (continuous), dialect group (Cantonese or Hokkien), educational level (no formal education, primary school, or secondary school or higher), body mass index (< 18.5, 18.5–22.9, 23.0-27.4, 27.5 + kg/m^2^), smoking status (never, former, or current), alcohol frequency (none, monthly, weekly, or daily), physical activity (< 0.5 h/wk, 0.5–3.9 h/wk, or ≥ 4 h/wk), sleep duration (< 6 h/d, 6–8 h/d, or > 8 h/d), history of hypertension (yes/no), history of diabetes (yes/no), history of cardiovascular disease (yes/no), and history of cancer (yes/no). The strata variable was not included in the model when stratifying by itself. For the subgroups of smoking status, non-smokers include never and ever smokers at baseline, and smokers mean current smokers at baseline

Subgroup analyses indicated that the association between UPF intake and the risk of all-cause mortality was stronger among non-smokers than smokers at recruitment (*P* for interaction = 0.03); the HR comparing extreme quintiles was 1.08 (95% CI, 1.03, 1.13) in non-smokers vs. 1.01 (95% CI, 0.94, 1.08) in smokers (Fig. 1). Sensitivity analyses were largely consistent when we excluded participants who died within 2 or 3 years or participants with CVD or cancer at baseline (Supplementary Table 2). The inclusion of whole-wheat bread and hard liquor individually and in combination did not change the results. The risk estimates for all the associations remained materially the same when we used quintiles based on the whole cohort (Supplementary Table 3), and with the absolute intake (g/d) instead of proportion for UPF intake (Supplementary Table 4).

### Associations between UPF subgroups intake and mortality

**Fig. 2:**
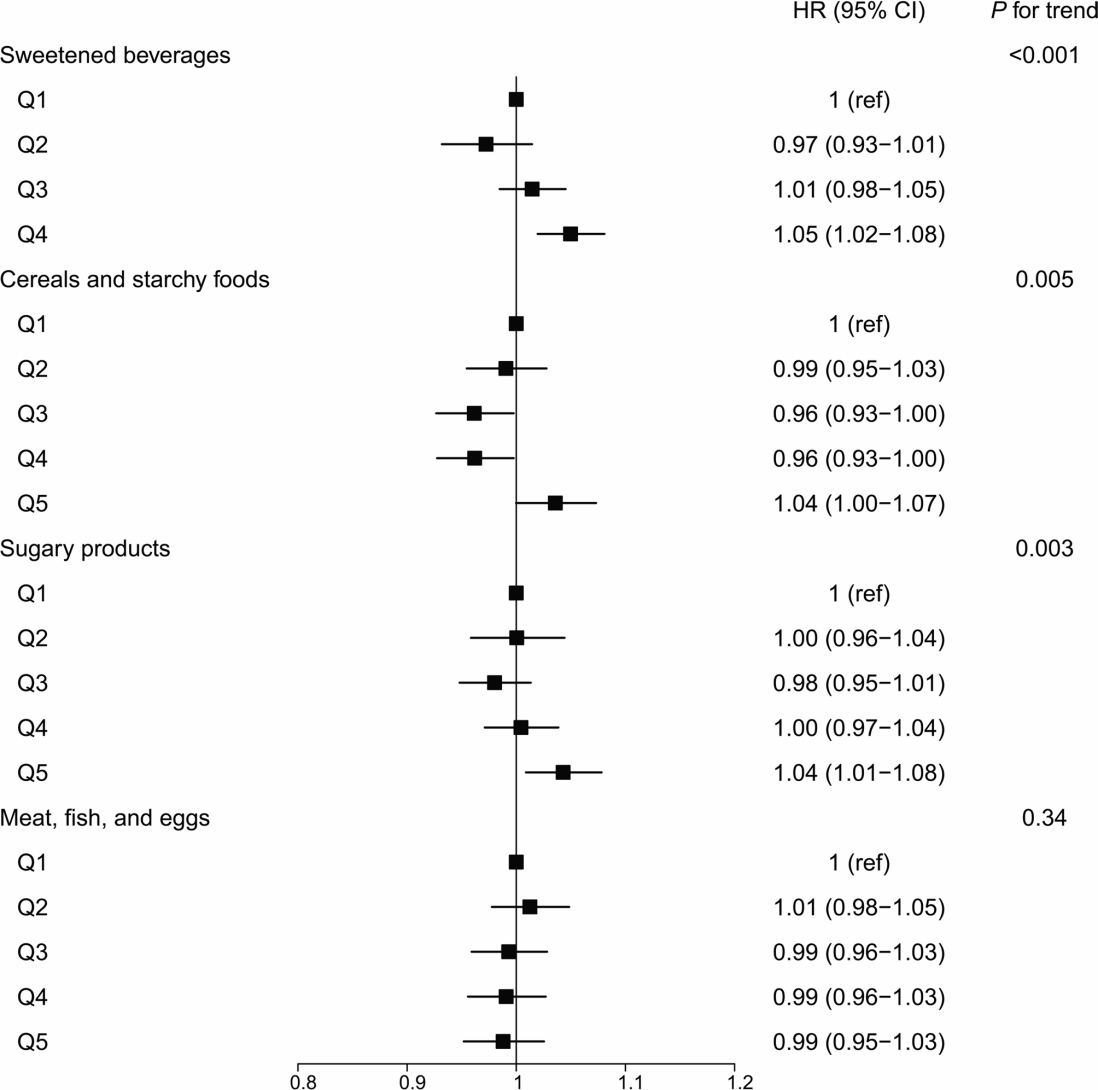
Hazard ratios (95% confidence intervals) for associations of main food subgroups of ultra-processed food intake with all-cause mortality in the Singapore Chinese Health Study. HR indicates hazard ratio. CI indicates confidence interval. Multivariable adjusted models were adjusted for age (continuous), sex (male or female), total energy intake (continuous), dialect group (Cantonese or Hokkien), educational level (no formal education, primary school, or secondary school or higher), body mass index (< 18.5, 18.5–22.9, 23.0-27.4, 27.5 + kg/m^2^), smoking status (never, former, or current), alcohol frequency (none, monthly, weekly, or daily), physical activity (< 0.5 h/wk, 0.5–3.9 h/wk, or ≥ 4 h/wk), sleep duration (< 6 h/d, 6–8 h/d, or > 8 h/d), history of hypertension (yes/no), history of diabetes (yes/no), history of cardiovascular disease (yes/no), and history of cancer (yes/no). The top four consumed subgroups of UPFs were focused. Given the weight ratio of sweetened beverages cannot be divided into quintiles, we divided it into quartiles

Among the top four consumed UPF subgroups, sweetened beverages and sugary products showed significant association with all-cause mortality. HRs (95% CIs) were 1.05 (95% CI, 1.02, 1.08) for sweetened beverages and 1.04 (95% CI, 1.01, 1.08) for sugary products in comparison between extreme quintiles (Fig. 2).

## Discussion

In this prospective study of Chinese adults living in Singapore, higher UPF intake was associated with higher risks of mortality from all-cause, CVDs, and respiratory diseases. The associations were still significant after accounting for diet quality and antioxidant capacity. The association with all-cause mortality was significant for intake of sweetened beverages and sugary products among the subgroups of UPFs. In stratified analysis, the UPF-mortality association was stronger among non-smokers than smokers.

A recently published meta-analysis included studies conducted in Western countries, comprising three in the US [27–29], two in Spain [30, 31], one in France [32], and one in Italy [33], and the results showed that the highest category group of UPF intake was associated with a higher risk of all-cause mortality compared with the lowest category group (HR 1.21; 95% CI, 1.15, 1.27) [10]. The Prospective Urban and Rural Epidemiology (PURE) study covered 7 regions that included countries in North America and Europe, South America, Africa, the Middle East, South Asia, South East Asia, and China, and observed that the highest UPF intake was associated with a 28% higher risk of all-cause mortality compared with the lowest intake in this global analysis [34]. However, the authors did not do subgroup analysis by region or country. As for Asian countries, to our best knowledge, only one Korean cohort investigated the association between total UPF intake and all-cause mortality and observed non-significant results [13], which contradicted the significant results in our study of Chinese in Singapore. Nonetheless, this Korean study still observed significant associations between higher intake of specific UPF subgroups (ultra-processed red meat and fish in both sexes, and milk and soymilk drinks in male) and increased risk of all-cause mortality [13]. Since different subgroups of UPFs may have different impacts on mortality, the conflict between our results and those from the aforementioned Korean study could be explained by differences in the intake of subgroups in both cohorts.

For cause-specific mortality, our findings of an association with CVD mortality were largely consistent with studies from the UK and Italy, which also observed that higher UPF intake was associated with a higher risk of CVD mortality [33, 35]. However, one Spain cohort and three US cohorts observed null associations with CVD mortality [29, 30, 36]. Our finding of a null association with cancer mortality was consistent with the null findings from three US cohorts [36, 37] but conflicted with the finding of significant results in a UK cohort [37]. These inconsistent results could, again, be explained by differences in the UPF subgroups in different populations. Few studies have investigated the associations with respiratory disease mortality. Consistent with our study, the Nurses’ Health Study and Health Professionals Follow-up Study also observed higher UPF intake was associated with an increased risk of respiratory disease mortality [36]. In contrast, the UK Biobank study did not observe a significant association between UPF intake and respiratory disease mortality [35], but we have noted that there were fewer respiratory disease deaths (793 events) in this study compared to our study (6,452 events).

Since higher UPF intake has been shown to be associated with reduced intake of plant-based foods, the association between UPFs and poor health could be confounded by a less healthful dietary pattern [38]. Indeed, a recent analysis of the Nurses’ Health Study and Health Professionals Follow-up Study observed that the UPF-mortality associations were null after adjusting for the AHEI score [36]. However, in our cohort, we still observed significant UPF-mortality associations after adjusting for diet quality using the AHEI score or the VCEAC score. Consistent with our finding, the majority of the associations of UPFs with health-related outcomes also remained significant and similar in magnitude after adjustment for diet quality or pattern [38, 39]. Of interest, the magnitude of the association between UPF intake and all-cause mortality was greater among non-smokers than smokers in our study, which was also shown in the Nurses’ Health Study and Health Professionals Follow-up Study [36]. Since non-smokers generally start with a lower baseline risk for mortality compared to smokers, the ill effects of UPFs on health could be more apparent in non-smokers than in smokers. However, this hypothesis of ours requires further validation in future studies.

There are several possible reasons for the association between UPF intake and mortality. First, thermal processing of foods or heat treatment could produce toxic chemicals such as advanced glycation end products (AGEs) in breads, cookies, and French fries. AGEs could activate downstream cell signals and form irreversible covalent complexes with proteins, and thus promote inflammation and oxidative stress [40], which are pivotal risk factors for cardiovascular and respiratory disease [41–44]. Second, additives such as emulsifiers are frequently used to make UPFs appealing and palatable. Evidence suggests that emulsifiers could alter the composition of gut microbiota, thus promoting low-grade inflammation and metabolic syndrome [45]. Metabolic syndrome is an independent risk factor for greater lung function impairment, worsening respiratory symptoms, and asthma [46]. Third, endocrine-disrupting chemicals such as bisphenol A could migrate from food packaging into food [47]. Epidemiological studies suggested that endocrine disruptors are associated with obesity and diabetes [48]. Bisphenol A could also influence the functions of lungs through stimulating metastasis and inducing lung fibrosis [49].

This study’s strengths included a large sample size, a long follow-up period, and its prospective design. Nevertheless, our study has several Limitations. First, the FFQ used in the SCHS was not specifically designed to collect dietary information according to the Nova classification; thus, we could have underestimated the measurement of UPF intake in this cohort. Second, the dietary information was only collected at baseline, and potential changes during follow-up might affect the magnitude of the results. Third, as our findings were derived from Chinese Living in Singapore, our findings may not be generalizable to other populations with vastly different compositions of UPFs in the daily diet. Fourth, given the observational design, despite comprehensive adjustment of covariates, residual confounding could not be completely ruled out. Fifth, the Nova classification is not fully objective since this food classification is not based on unequivocal, distinct physicochemical aspects of foods. Caution is needed in generalizing these findings. Finally, considering that our dietary data was collected from 1993 to 1998, more studies with more recent dietary data are needed to explore the association between UPF intake and mortality in Asian populations.

## Conclusion

In this prospective study of Singapore Chinese adults, higher UPF intake was associated with higher risks of mortality from all-cause, CVDs, and respiratory diseases. Our findings showed that sweetened beverages (e.g. soft drinks) and sugary products (e.g. crackers and western cakes) could be major subgroups contributing to the harmful association of UPFs with mortality, and this suggests that it could be feasible and beneficial to focus on reducing the consumption of these subgroups in this Asian population. More studies are needed to identify the major harmful subgroups of UPFs in other Asian populations.

## Supplementary Information

Supplementary Material 1.

## Data Availability

Data described in the article, code book, and analytic code will be made available upon reasonable request.

## Abbreviations

AHEI: Alternative Healthy Eating Index
BMI: Body mass index
CI: Confidence interval
CVD: Cardiovascular disease
FFQ: Food frequency questionnaire
HR: Hazard ratio
PURE: Prospective Urban and Rural Epidemiology
RCS: Restricted cubic spline
SCHS: Singapore Chinese Health Study
UPF: Ultra-processed food
US: United States
UK: United Kingdom
VCEAC: Vitamin C Equivalent Antioxidant
