# Supplementary material for: Consumption of ultra-processed foods and risk of all-cause and cause-specific mortality: the Singapore Chinese health study
Source: Nutr J. 2025 Sep 29;24:146. doi: 10.1186/s12937-025-01219-0 (PMC12481944; doi:10.1186/s12937-025-01219-0)
Supplement: Supplementary file 1 — Supplementary Material 1. [file 12937_2025_1219_MOESM1_ESM.docx]

**Consumption of ultra-processed foods** **and risk of all-cause and cause-specific mortality:** **the Singapore Chinese Health Study**

Yue Li et al.

**Online Supplementary Material**

**Supplementary Table 1.** **Food products considered as** **ultra-processed foods according to** **the NOVA classification.**

| Ultra-processed food group^a^ | Examples of food |
| --- | --- |
| Sweetened beverages | Soft drinks, Milo, Ovaltine, and Horlicks |
| Dairy based products | Yakult, Vitagen, ice cream, and frozen yogurt |
| Cereals and starchy foods | Hot oats or other hot cereals, cornflakes or other cold cereals, white sliced bread, and hotcake |
| Sugary products | Crackers and biscuits, western cakes, and coconut desserts |
| Savory snacks | Hamburger and cheeseburger, French fries, sandwiches, pizza, and baked buns with processed meat |
| Meat, fish, and eggs | Deep-fried chicken, sausage, ham, hot dogs, luncheon meat, deep-fried fish, and preserved eggs |
| Spread and sweetener | Margarine, peanut butter, and artificial sweetener |

^a^ Since hard liquor is classified as UPFs, but alcohol is a well-investigated risk factor for mortality, we did not consider hard liquor in UPFs in our primary analysis. Since whole-wheat bread, which belongs to UPFs, has been associated with lower ischemic heart disease mortality in our study population, we did not include whole-wheat bread in our primary analysis.

**Supplementary Table 2.** **Sensitivity analysis for associations of ultra-processed food intake with** **mortality in the Singapore Chinese Health Study.**^a^

|  | All-cause mortality |  | CVD mortality |  | Cancer mortality |  | Respiratory disease mortality |
| --- | --- | --- | --- | --- | --- | --- | --- |
| Ultra-processed food subgroups | HR (95% CI)^b^ |  | HR (95% CI)^b^ |  | HR (95% CI)^b^ |  | HR (95% CI)^b^ |
| Exclusion of within 2 years of follow up | 1.03 (1.01-1.04) |  | 1.03 (1.01-1.06) |  | 0.99 (0.97-1.02) |  | 1.06 (1.03-1.09) |
| Exclusion of within 3 years of follow up | 1.03 (1.01-1.04) |  | 1.03 (1.01-1.06) |  | 0.99 (0.97-1.02) |  | 1.05 (1.02-1.09) |
| Exclusion of prevalent CVD or cancer at baseline | 1.02 (1.01-1.04) |  | 1.03 (1.00-1.05) |  | 0.99 (0.96-1.01) |  | 1.06 (1.03-1.10) |
| Include whole-wheat bread in the total UPF definition | 1.03 (1.02-1.05) |  | 1.04 (1.02-1.07) |  | 1.00 (0.97-1.03) |  | 1.06 (1.03-1.09) |
| Include hard liquor in the total UPF definition | 1.04 (1.02-1.05) |  | 1.05 (1.02-1.07) |  | 1.00 (0.98-1.03) |  | 1.06 (1.03-1.09) |
| Include whole-wheat bread and hard liquor in the total UPF definition | 1.03 (1.02-1.05) |  | 1.04 (1.02-1.07) |  | 1.00 (0.97-1.03) |  | 1.06 (1.02-1.09) |

HR: hazard ratio; CI: confidence intervals; CVD: cardiovascular disease; UPF: ultra-processed food.

^a^ All model was adjusted for age (continuous), sex (male or female), total energy intake (continuous), dialect group (Cantonese or Hokkien), educational level (no formal education, primary school, or secondary school or higher), body mass index (<18.5, 18.5-22.9, 23.0-27.4, 27.5+ kg/m^2^), smoking status (never, former, or current), alcohol frequency (none, monthly, weekly, or daily), physical activity (<0.5 h/wk, 0.5-3.9 h/wk, or ≥4 h/wk), sleep duration (<6 h/d, 6-8 h/d, or >8 h/d), history of hypertension (yes/no), history of diabetes (yes/no), history of cardiovascular disease (yes/no), and history of cancer (yes/no).

^b^ Hazard ratio for per increase of 10% in the proportion of ultra-processed food intake.

**Supplementary Table 3.** **Hazard ratios (95% confidence intervals) for associations of u****ltra-processed food intake (****quintiles based on whole cohort) with** **mortality in the Singapore Chinese Health Study.**

|  | Quintiles of ultra-processed food weight, % | | | | |  |
| --- | --- | --- | --- | --- | --- | --- |
|  | Q1 | Q2 | Q3 | Q4 | Q5 | *P* trend |
| Median (IQR) | 1.1 (0.5-1.6) | 3.3 (2.7-3.9) | 6.1 (5.3-7.0) | 11.0 (9.5-12.9) | 20.3 (17.3-24.8) |  |
| All-cause mortality |  |  |  |  |  |  |
| Cases/person-years | 6385/267563 | 5788/275102 | 5565/276460 | 5554/274628 | 6180/265267 |  |
| Full adjusted Model^a^ | 1 (ref) | 0.97 (0.94-1.01) | 1.00 (0.96-1.03) | 1.03 (1.00-1.07) | 1.06 (1.02-1.10) | <0.001 |
| CVD mortality |  |  |  |  |  |  |
| Cases/person-years | 1974/267563 | 1840/275102 | 1712/276460 | 1782/274628 | 2014/265267 |  |
| Full adjusted Model^a^ | 1 (ref) | 0.98 (0.92-1.05) | 0.97 (0.91-1.04) | 1.06 (0.99-1.13) | 1.08 (1.01-1.15) | <0.001 |
| Cancer mortality |  |  |  |  |  |  |
| Cases/person-years | 2105/267563 | 1847/275102 | 1802/276460 | 1698/274628 | 1807/265267 |  |
| Full adjusted Model^a^ | 1 (ref) | 0.96 (0.90-1.02) | 1.00 (0.94-1.06) | 0.96 (0.90-1.03) | 1.00 (0.94-1.07) | 0.78 |
| Respiratory disease mortality |  |  |  |  |  |  |
| Cases/person-years | 1351/267563 | 1257/275102 | 1219/276460 | 1262/274628 | 1363/265267 |  |
| Full adjusted Model^a^ | 1 (ref) | 1.00 (0.93-1.08) | 1.04 (0.96-1.12) | 1.13 (1.04-1.22) | 1.08 (1.00-1.17) | 0.006 |

IQR: interquartile range; CVD: cardiovascular disease.

^a^ Adjusted for age (continuous), sex (male or female), total energy intake (continuous), dialect group (Cantonese or Hokkien), educational level (no formal education, primary school, or secondary school or higher), body mass index (<18.5, 18.5-22.9, 23.0-27.4, 27.5+ kg/m^2^), smoking status (never, former, or current), alcohol frequency (none, monthly, weekly, or daily), physical activity (<0.5 h/wk, 0.5-3.9 h/wk, or ≥4 h/wk), sleep duration (<6 h/d, 6-8 h/d, or >8 h/d), history of hypertension (yes/no), history of diabetes (yes/no), history of cardiovascular disease (yes/no), and history of cancer (yes/no)

**Supplementary Table 4.** **Hazard ratios (95% confidence intervals) for associations of ultra-processed food by weight with mortality in the Singapore Chinese Health Study.**

|  | Sex-specific quintiles of ultra-processed food weight, g/d | | | | |  |  |
| --- | --- | --- | --- | --- | --- | --- | --- |
|  | Q1 | Q2 | Q3 | Q4 | Q5 | *P* trend | Continuous^a^ |
| Median (IQR) | 18.0 (7.9-26.2) | 54.7 (44.9-63.2) | 103.7 (89.3-120.0) | 204.2 (166.5-253.7) | 350.7 (310.6-453.1) |  |  |
| All-cause mortality |  |  |  |  |  |  |  |
| Cases/person-years | 6287/268367 | 5968/272796 | 5539/276192 | 5697/272586 | 5981/269080 |  |  |
| Full adjusted Model^b^ | 1 (ref) | 1.01 (0.97-1.04) | 0.99 (0.95-1.02) | 1.04 (1.00-1.07) | 1.07 (1.03-1.11) | <0.001 | 1.03 (1.02-1.04) |
| CVD mortality |  |  |  |  |  |  |  |
| Cases/person-years | 1999/268367 | 1876/272796 | 1734/276192 | 1781/272586 | 1932/269080 |  |  |
| Full adjusted Model^b^ | 1 (ref) | 0.98 (0.92-1.05) | 0.97 (0.91-1.03) | 1.01 (0.95-1.08) | 1.08 (1.01-1.15) | 0.004 | 1.04 (1.02-1.06) |
| Cancer mortality |  |  |  |  |  |  |  |
| Cases/person-years | 1993/268367 | 1890/272796 | 1800/276192 | 1762/272586 | 1814/269080 |  |  |
| Full adjusted Model^b^ | 1 (ref) | 1.00 (0.94-1.07) | 1.00 (0.94-1.07) | 1.00 (0.94-1.07) | 1.02 (0.96-1.10) | 0.49 | 1.00 (0.98-1.02) |
| Respiratory disease mortality |  |  |  |  |  |  |  |
| Cases/person-years | 1330/268367 | 1318/272796 | 1193/276192 | 1291/272586 | 1320/269080 |  |  |
| Full adjusted Model^b^ | 1 (ref) | 1.04 (0.96-1.12) | 0.99 (0.92-1.08) | 1.11 (1.02-1.20) | 1.10 (1.01-1.19) | 0.007 | 1.04 (1.02-1.07) |

IQR: interquartile range; CVD: cardiovascular disease.

^a^ Calculated per standard deviation increment in ultra-processed foods.

^b^ Adjusted for age (continuous), sex (male or female), total energy intake (continuous), dialect group (Cantonese or Hokkien), educational level (no formal education, primary school, or secondary school or higher), body mass index (<18.5, 18.5-22.9, 23.0-27.4, 27.5+ kg/m^2^), smoking status (never, former, or current), alcohol frequency (none, monthly, weekly, or daily), physical activity (<0.5 h/wk, 0.5-3.9 h/wk, or ≥4 h/wk), sleep duration (<6 h/d, 6-8 h/d, or >8 h/d), history of hypertension (yes/no), history of diabetes (yes/no), history of cardiovascular disease (yes/no), and history of cancer (yes/no).

Participants recruited at baseline with FFQ (n= 63257)

Extreme energy intake (male: total energy<700; >3700 kcal/day; female: total energy<600; >3000 kcal/day); (n = 1060)

Participants with reasonable energy intake (n = 62197)

**Supplementary Figure 1. Selection of study participants.**


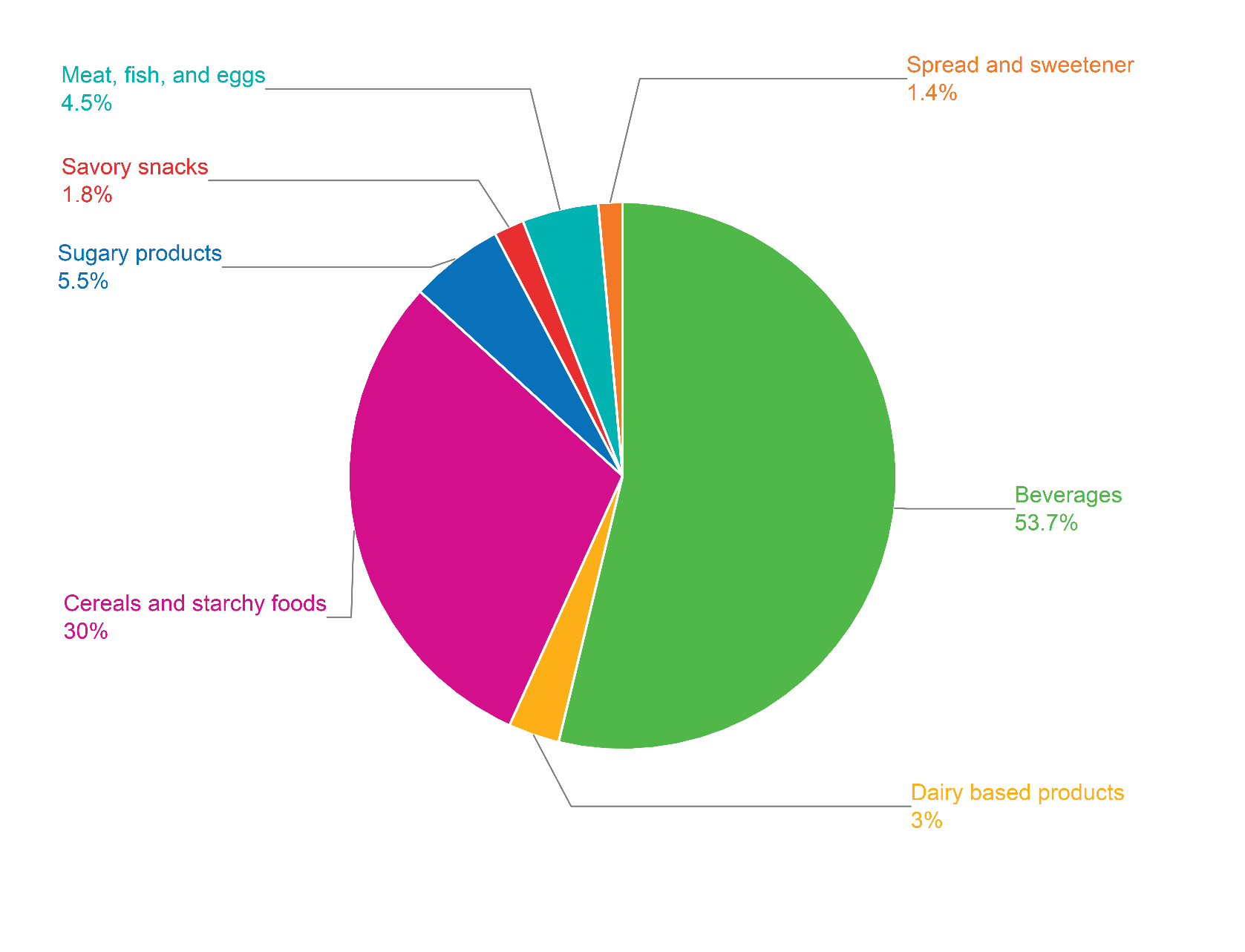


**Supplementary Figure 2.** **Relative contribution (%) of each food subgroup to consumption of ultra-processed foods in the diet.**


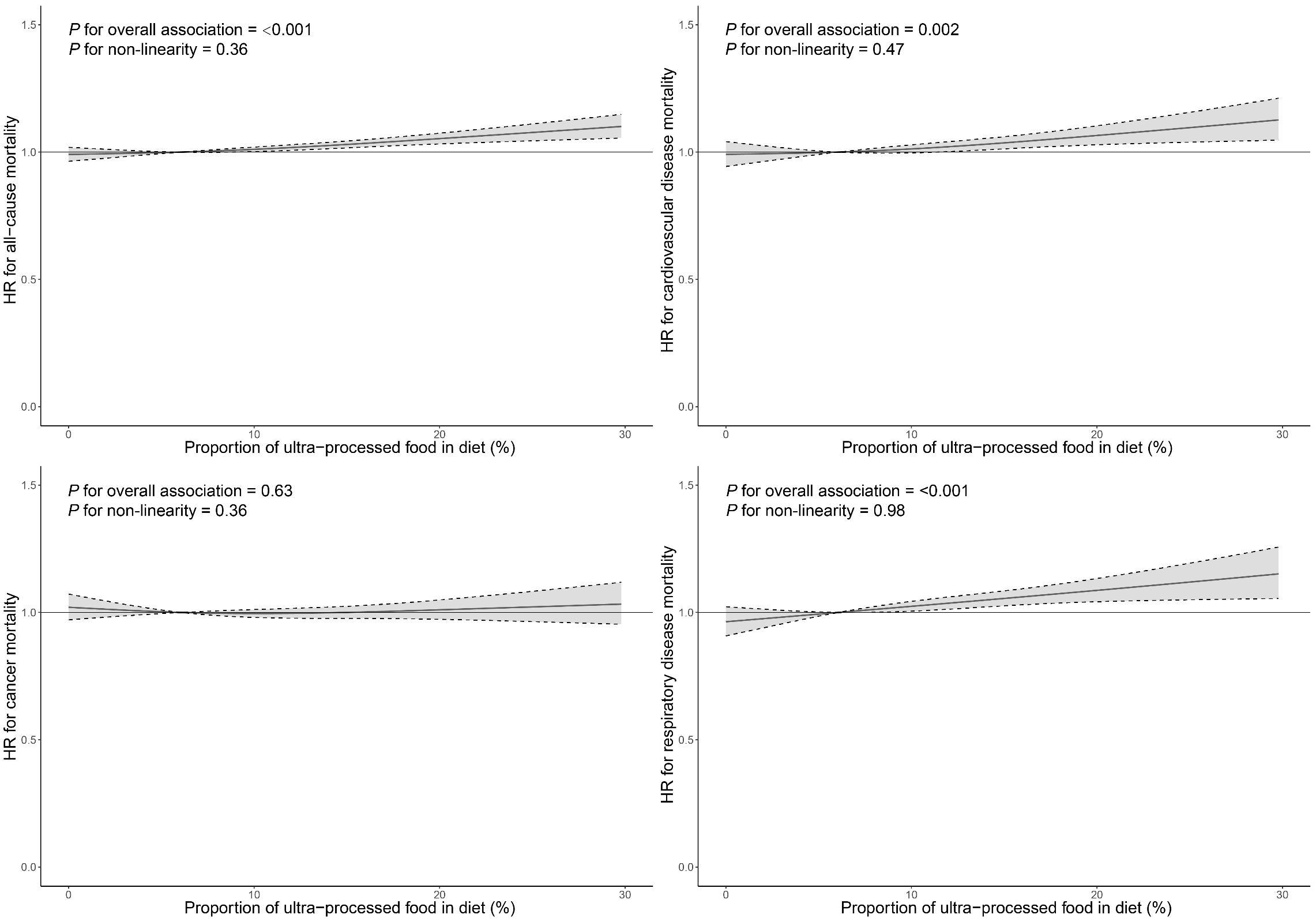


**Supplementary Figure 3. Restricted cubic spline analysis of ultra-processed food consumption with all-cause mortality, cardiovascular disease mortality, cancer mortality, and respiratory disease mortality in the Singapore Chinese Health Study.**

HRs with 95% confidence intervals (CIs) were calculated based on the multivariable model adjusted for age (continuous), sex (male or female), total energy intake (continuous), dialect group (Cantonese or Hokkien), educational level (no formal education, primary school, or secondary school or higher), body mass index (<18.5, 18.5-22.9, 23.0-27.4, 27.5+ kg/m^2^), smoking status (never, former, or current), alcohol frequency (none, monthly, weekly, or daily), physical activity (<0.5 h/wk, 0.5-3.9 h/wk, or ≥4 h/wk), sleep duration (<6 h/d, 6-8 h/d, or >8 h/d), history of hypertension (yes/no), history of diabetes (yes/no), history of cardiovascular disease (yes/no), and history of cancer (yes/no). The reference values for HRs were set as 6.1% (the median value of the proportion of ultra-processed food intake). Three knots were located at the 10th, 50th, and 90th percentiles of the exposure. The gray zones indicated 95% CIs.
